# Challenges and opportunities of vaccination during pregnancy: perspectives of 20 healthcare professionals

**DOI:** 10.1057/s41271-025-00548-y

**Published:** 2025-01-22

**Authors:** Mohammad S. Razai, Sally Hargreaves, Pippa Oakeshott

**Affiliations:** 1 Primary Care Unit, Department of Public Health and Primary Care, University of Cambridge, East Forvie Building, Forvie Site, Robinson Way, Cambridge, CB2 0SR UK; 2 George’s School of Health and Medical Sciences, Population Health Research Institute, City St George’s, University of London, London, UK; 3 Institute for Infection and Immunity, The Migrant Health Research Unit, City St George’s University of London, London, UK

**Keywords:** Healthcare professional, Maternal vaccinations, Health policy, Vaccine uptake, Technology, Artificial intelligence

## Abstract

Vaccination during pregnancy is crucial due to increased maternal vulnerability to infectious diseases. However, uptake of recommended vaccines (influenza, pertussis, COVID-19) remains suboptimal, particularly among disadvantaged groups. This qualitative study explored healthcare professionals’ (HCPs) perspectives, selected purposively, on factors influencing maternal vaccination in London. Data from a workshop transcript and an online quality improvement survey involving 15 GPs, two midwives, two obstetricians and one pharmacist were thematically analysed. HCPs highlighted that pregnant women perceive the benefits of pertussis vaccination more positively than other vaccines. Clear, consistent communication and integrating vaccinations into routine antenatal care were identified as essential for improving accessibility and convenience. The critical role of midwives influencing vaccination decisions was emphasised. While recognising the potential of AI-based technologies to disseminate vaccine information, concerns were raised about trust, digital literacy and information quality, highlighting the need for tailored, reliable strategies to boost maternal vaccination rates and improve health outcomes.

**Supplementary Information:**

The online version contains supplementary material available at 10.1057/s41271-025-00548-y.

## Key messages

- Embedding maternal vaccinations (influenza, pertussis, COVID-19 and the newly recommended RSV) into routine antenatal care, such as during scans or check-ups, improves accessibility and convenience, likely increasing vaccine uptake.
- Midwives play a crucial role in maternal vaccination decisions. Consistent, clear, unambiguous messaging from midwives can build trust and drive higher vaccination rates among pregnant women.
- Addressing vaccine hesitancy requires targeted strategies, including personalised communication and cautious use of AI-powered digital tools, ensuring they provide accurate, unbiased information to diverse populations.

## Introduction

The necessity of vaccination during pregnancy, a period characterised by increased vulnerability to infectious diseases, cannot be overstated [1, 2]. Vaccines not only protect the expectant mother but also confer immunity to the unborn child, offering a shield against potential infections during the earliest phase of life [2]. Therefore, uptake of influenza, pertussis and COVID-19 vaccinations during pregnancy is strongly recommended [3]. Additionally, from September 2024, the UK recommends the Respiratory Syncytial Virus (RSV) vaccine for pregnant women from 28 weeks’ gestation to protect newborns against severe respiratory illnesses [4]. Despite these recommendations, vaccination rates among pregnant women remain suboptimal in England, with rates reported as 32% for influenza in 2024, 58% for pertussis in 2023 and 59% for COVID-19 in 2022 [5–7], demonstrating a gap between public health guidelines and actual practice [1, 8, 9].

Several factors contribute to this gap. Misinformation, mistrust and concerns about vaccine safety, particularly heightened during the COVID-19 pandemic, have amplified vaccine hesitancy [10]. The influence of social networks, healthcare professionals’ advice and cultural beliefs also play crucial roles in shaping vaccination decisions [8]. Moreover, systemic barriers such as access to healthcare services and socio-economic factors further complicate efforts to improve vaccination uptake [11, 12].

Rapid technological advancements, particularly the emergence of artificial intelligence (AI) and digital health tools, present opportunities to enhance public health messaging and foster better patient engagement [13, 14], including vaccine uptake. These developments could transform how information is disseminated and how healthcare providers interact with patients during antenatal care.

This study aimed to explore the perspectives of healthcare professionals (HCPs) on the opportunities and challenges of maternal vaccinations and offer a deeper understanding of the factors that impact vaccination uptake among pregnant women. In 2023–24, MSR conducted a workshop and an online survey of 20 UK-based HCPs.

## Data and methods

### Study design

This study employed a qualitative research design, focusing on a thematic analysis of a workshop transcript and an online survey (Table S1). The workshop (conducted in September 2023) and the survey (September to October 2023) brought together diverse stakeholders, including healthcare professionals and researchers from Greater London area. The primary aim was to explore HCPs’ perspectives on factors influencing vaccination uptake among pregnant women.

## Data collection

The data for this study were collected through a structured workshop comprising a series of guided discussions (Table S2) and an online survey (Table S1). The workshop was audio-recorded with the consent of all participants. Key topics included vaccination trends, communication strategies, the role of healthcare professionals, the impact of digital tools and barriers to vaccine uptake among pregnant women. The workshop methodology adopted for this study leverages interactive, participant-centred learning to engage healthcare professionals in the critical examination of barriers to maternal vaccinations and the identification of actionable strategies to increase vaccine uptake. This approach is grounded in adult learning theory, which posits that adults learn best through experiential techniques that involve active participation and reflection on the learning process [15]. Workshops facilitate a dynamic exchange of ideas, encourage collaborative problem solving and enable immediate application of theoretical concepts to real-world scenarios. This method emphasises dialogue and critical thinking as pivotal to transformative learning [16]. By employing a workshop format, the study gathered rich qualitative data on perceived barriers and potential strategies from those directly involved in the delivery of maternal healthcare and contributed to the professional development of participants by engaging them in reflective practice and peer learning. This methodology is particularly suited to exploring complex behavioural and systemic issues in healthcare settings, where the perspectives and expertise of frontline workers are invaluable for informing effective interventions [17].

An 8-point semi-structured quality improvement questionnaire survey was disseminated online to a group of healthcare professionals, with questions focusing on barriers to maternal vaccinations and strategies to enhance vaccine uptake. The development of the questionnaire was guided by principles of quality improvement, ensuring that its questions were both relevant and effective for the study’s objectives. The survey was piloted before dissemination.

## Participants

Participants were selected through a purposive sampling to ensure a wide range of perspectives. The sampling criteria focused on professional experience in healthcare, expertise in public health or obstetrics, experience in healthcare policy, or direct experience with antenatal care. The participants’ demographic details (age, gender and professional background) were recorded to contextualise the responses. Participants were from urban or suburban healthcare settings but not rural. The survey was forwarded as an anonymous MS Forms link to obstetricians, midwives and pharmacists in South London. Respondents accessed the survey through this link. However, the exact number of recipients is unknown, making it impossible to estimate the denominator or response rate accurately. Workshop attendees were selected through convenience sampling from a monthly academic GP mailing list. An invitation email was sent to GPs on this list, inviting them to participate in the workshop.

## Data processing and thematic analysis

The anonymised workshop audio recordings were transcribed verbatim by a professional transcription service. Data were coded in MS Word and Excel for organisation and analysis. The free-text survey responses underwent a coding process similar to the workshop transcript.

The analysis followed Braun and Clarke’s six-phase process of thematic analysis [18], involving

1. Familiarisation with the data: Initial reading of the transcripts to gain an in-depth understanding of the content.
2. Generating initial codes: Systematically coding the dataset in a way that tags specific data features relevant to the research questions.
3. Searching for themes: Collating codes into potential themes and gathering all data relevant to each theme.
4. Reviewing themes: Checking if the themes work in relation to the coded extracts and the entire dataset, generating a thematic map of the analysis.
5. Defining and naming themes: Further refining the specifics of each theme and generating clear definitions and names for each theme.
6. Producing the report: The final step involved the selection of compelling extract examples, the final analysis of the data and the production of the report**.**

## Results

We recruited 20 participants: 15 GPs/family doctors and GP trainees aged 28 to 72 with diverse backgrounds participated in the workshop. Five other HCPs (two midwives, two obstetricians and one pharmacist) responded to the survey. Most participants were female (15/20, 75%) and around half (9/20, 45%) self-identified as ethnic minorities. They had worked as healthcare professionals (GPs, midwives, obstetricians and pharmacists) for 2 to 30 years and were engaged in patient-facing hospitals and general practices in urban and suburban settings. None worked in rural areas, and none had direct experience of using artificial intelligence in healthcare.

The following themes emerged from the workshop and survey responses.

### Vaccination uptake trends and influences

This theme encompasses the dynamic and evolving nature of vaccination uptake over time, highlighting the impact of external factors such as global health crises (e.g. the COVID-19 pandemic) and changes in public sentiment towards vaccines. Participants observed that while initial responses to specific vaccination campaigns were positive, this enthusiasm waned over time for various reasons, including growing vaccine hesitancy (#1 in Table 1).

**Table 1:** Direct quotation from 20 healthcare professionals who participated in this study

Citation #	Quotation	References
1	Initially uptake was very good… That has dwindled… probably has taken a big hit since covid	Participant 12
2	People are generally very positive about [whooping cough vaccine] … The messaging around that is quite clear… It’s about protecting the baby	Participant 3
3	Pertussis is established now… You’re protecting your baby whereas with Covid it’s a bit more vague.. Like [saying] we hope it will protect you, but we’re not sure, and we don’t really know what the side effects will be because we don’t have long-term studies on it yet	Participant 6
4	You know that the whooping cough vaccine is so clearly included in all of your antenatal booklets and it’s marked and it says make sure you get it by this week. And they've got the walk-in clinic upstairs. So it makes it very easy and very clear. Covid and flu doesn’t appear in the booklet, so coming from a patient perspective, it’s less clear and I think you have to make more of an effort to try and seek it out	Participant 5
5	Mine was linked to one of my scans. They had a clinic running, so you’re right there, and they’re offering [vaccines], and it feels part of your antenatal care rather than something extra that you’re having to make a decision about	Participant 7
6	Clinic booking visits would be quite key, so everything is laid out. This is what’s going to happen. It'll be this many weeks pregnant when you will have this vaccination	Participant 14
7	Groups who don’t understand vaccination… there’s trust… and knowledge that some groups don’t have… And I think those are really messy problems to try and solve…Having trusted people from the communities from which people come, who are advocates, might be helpful as well	Participant 11
8	Advice coming from midwives is better than coming from doctors. They [pregnant women] have a bit more time with midwives. It’s not the continuity that there used to be, but I feel like that’s often a better source than doctors	Participant 19
9	What are midwives’ opinions about covid vaccines and flu vaccines compared to pertussis, where I know they’re overwhelmingly positive and enthusiastic? If they’re [midwives] the people to lead on it, it’s really important that their messaging is consistent	Participant 3
10	I changed midwife teams… The community midwife I am under sends lots of information and emails about vaccines and additional links. The one I was under before didn’t. So already, within one hospital, patients are getting very different information. So it could be a really nice way of standardising the information people receive from the get-go	Participant 13
11	We need trustworthy staff offering the vaccine at every contact ie consistency of the importance on the maternal vaccinations; timeliness of vaccines; accessibility; dedicated vaccination team; good staff knowledge	Participant 18
12	Using AI for information sharing with patients… It depends on the quality of the information. But I guess the issue with AI is making sure that it speaks to all of the groups who it needs to speak to. And obviously that the information is correct. It should not be succumbing to commercial interests or other biases that might be inherent in the stuff that it’s using to produce its answer	Participant 16
13	I'm involved in a postnatal blood pressure intervention, and they've been using an AI just to like design, resources for women… They presented us with three different versions of some kind of instructions and background, not telling us who wrote them. And all of the patients and all of the clinicians picked the one that AI generated…That’s quite a different way of involving AI	Participant 18
14	AI is designed to create something plausible but not necessarily true. So that feels like a real danger when you start using it for this sort of thing	Participant 17
15	Some populations won’t necessarily be tech-savvy… But I think if there’s an ease of [access] and the information is only coming from non-commercial and NHS Choices or prepared leaflets, that might work	Participant 6
16	Will people trust chatbots and that kind of AI? I don’t know about pregnancy, women are so nervous about vaccines. Is that going to be a benefit or is it just going to lead to them still wanting to see the GP or the midwife to talk about it anyway?	Participant 4
17	Do you think there’s a kind of adverse consequence potentially, which is disengaging people from healthcare professionals? It could increase distrust. So if you withdraw people from midwifery visits and direct them towards a chat bot for their queries, it just becomes like another Instagram page… That could be pro, could be against. What’s it doing to build trust between patient and the healthcare system?	Participant 15

### Differences in vaccine acceptance

Participants discussed how acceptance levels vary across different vaccines. They noted that the pertussis/whooping cough vaccine was more widely accepted by pregnant women due to clearer communication about their direct benefits, especially for vulnerable groups like infants.

This contrast highlights the importance of how vaccines are presented to the public and their perceived benefits (#2 and #3 in Table 1).

### Communication and messaging

The importance of effective communication strategies in shaping public opinion on vaccination was a prominent theme. Participants emphasised the need for clear and consistent, messages. The discussion acknowledged current challenges in communication, particularly related to the various benefits of the different vaccines (#4 in Table 1).

### Accessibility and convenience

Accessibility and convenience of vaccine services were identified as key determinants of uptake. The discussion highlighted the effectiveness of integrating vaccination services into existing healthcare routines, such as during antenatal scans or check-ups. This approach addresses logistical barriers and makes vaccination more seamless in healthcare (#5 and #6 in Table 1).

### Addressing marginalised groups

The responses underlined the importance of reaching out to marginalised groups who face unique barriers to vaccination, such as language barriers, lack of trust or limited access to reliable information. Tailoring strategies to these groups was seen as crucial for ensuring equitable vaccine coverage and tackling ethnic health disparities (#7 in Table 1).

### Role of healthcare professionals

Trust in healthcare professionals, especially midwives, emerged as a crucial factor in vaccination decisions. The discussion highlighted the influential role of these professionals in guiding expectant mothers through their vaccination choices. The continuity of care and trust associated with midwives were seen as more effective than general advice from other healthcare providers (#8, #9, #10 and #11 in Table 1).

### The potential of AI and digital tools

There were mixed opinions on integrating artificial intelligence and digital tools in vaccine education. Some participants saw great potential in using these technologies for disseminating accurate information and engaging patients, while others raised concerns about the quality, trust and personalisation of such digital interventions (#12, #13 and #14 in Table 1). There were concerns about the varying levels of technological and digital literacy among patients and how this impacts the effectiveness of digital tools (#15, #16 and #17 in Table 1).

## Discussion

This study illuminates key aspects of vaccination decision making among pregnant women, as perceived by 20 healthcare professionals (HCPs) through a workshop and an online survey. These HCPs shared a nuanced understanding of how factors such as communication, healthcare professional influence, accessibility, digital access, trust and health literacy play pivotal roles in shaping maternal attitudes and behaviours towards vaccination. The HCPs emphasised the need for clear and consistent messaging on maternal vaccination, underscoring the need for effective public health communication strategies. Additionally, these HCPs highlighted the influence of healthcare professionals, especially midwives, as a crucial determinant, highlighting their role as critical sources of information and guidance. These findings reflect the views and experiences of the participating HCPs and suggest the importance of tailored strategies to improve vaccination uptake among pregnant women.

The themes identified align with existing literature emphasising the importance of effective communication and trust in healthcare providers in vaccine decision making [1, 2, 8, 19]. Our findings also offer a deeper exploration of the HCPs’ perception of the role of midwives, expanding on the previous research on the influence of healthcare professionals [20]. Moreover, our study explores the HCPs’ views of the challenges and potential of digital health tools in vaccine communication. Recent evidence suggests that while GPs acknowledge the potential of AI-based technologies in primary care, they express concerns regarding the evidence base, accountability, bias, and increased workload, highlighting the need for rigorous evaluation of these technologies [21]. Vaccine hesitancy among healthcare professionals, attributed to concerns over vaccine safety, efficacy, necessity and access barriers, is well known [22]. However, despite reports of provider support for maternal vaccinations, uptake remains suboptimal [23]. The perspectives of HCPs in this study sheds light on the multifaceted challenges and complexities surrounding maternal vaccination, indicating that factors beyond vaccine hesitancy among pregnant women and healthcare providers contribute to low vaccination rates. These include effective messaging and communication strategies to enable informed decision making.

## Strengths and weaknesses

The study’s primary strength lies in its qualitative approach, offering in-depth insights into complex behaviours and attitudes that quantitative data alone might not reveal. The diversity of workshop participants provided a wide range of perspectives, enriching the analysis with varied experiences and expertise. To our knowledge, this study is the first study of HCP opinions of AI technologies, such as chatbots, in the context of vaccinating pregnant women.

However, this was a small study and may not capture the full spectrum of opinions and experiences across different regions or demographics of UK healthcare professionals. The findings are also subject to the inherent biases of the participants and the potential influence of group dynamics on individual responses. The recruitment method for the workshop participants relied on the willingness and availability of GPs to attend, which can introduce selection bias. Convenience sampling, while practical, may not fully capture the diversity and breadth of perspectives within the GP community.

It was not possible to provide a response rate for the survey. This lack of precise distribution data is a limitation, as it hinders the ability to determine the representativeness and generalisability of the survey results. Without knowing the total number of potential respondents, assessing the response rate and understanding the survey’s reach and potential bias in the sample is challenging. The limited inclusion of midwives in the study is a notable concern, particularly given the patients’ views on their central role in maternal healthcare and vaccination. Midwives are often key sources of information and support for pregnant women and their perspectives are crucial for understanding the factors influencing vaccination uptake. The reasons for their limited participation could include scheduling conflicts, lack of awareness of the study, or lower engagement with the recruitment methods used. Addressing this gap is essential for a comprehensive understanding of the barriers and facilitators to vaccination among pregnant women.

## Implications for practice

The findings highlight important implications for practice, such as:

- Enhanced communication strategies: healthcare authorities and practitioners must develop clearer, more targeted communication strategies, especially for vaccines during pregnancy.
- Empowering healthcare professionals: training and resources should be provided to midwives and other healthcare professionals to support them as key influencers in vaccination decisions. This includes effective communication training, such as preparing patients for vaccine discussions and ensuring they have the appropriate mindset to engage meaningfully. Practical and human-level factors, such as patients feeling rushed or delayed for their appointment, should also be considered, as these can impact the quality of the interaction.
- Tailoring digital health interventions: there is a need for careful design and implementation of digital health tools, ensuring they are accessible and reliable.

By tackling these areas, healthcare systems can make significant strides in improving vaccination uptake among pregnant women, including for the newly approved RSV vaccine, thereby enhancing maternal and neonatal health outcomes.

## Conclusion

By exploring the perspectives of diverse HCPs, this study provides a deeper understanding of the complex factors influencing vaccination behaviour among pregnant women and suggests possible avenues for improvement and change of practice.

## Supplementary Information

Below is the link to the electronic supplementary material.

Supplementary file1 (DOCX 142 KB)

## Data Availability

All data relevant to the study are available within the paper. However, raw data cannot be made publicly available because the data contains information that could compromise participants’ privacy and consent.
