# Supplementary material for: Challenges and opportunities of vaccination during pregnancy: perspectives of 20 healthcare professionals
Source: J Public Health Policy. 2025 Jan 22;46(2):411–22. doi: 10.1057/s41271-025-00548-y (PMC12119357; doi:10.1057/s41271-025-00548-y)
Supplement: Supplementary file 1 — Supplementary file1 (DOCX 142 KB) [file 41271_2025_548_MOESM1_ESM.docx]

**Journal of Public Health Policy**

Supplementary Materials

**Challenges and opportunities of vaccination during pregnancy: perspectives of 20 healthcare professionals**

**Running title:** Vaccination in pregnancy

Mohammad S Razai ^1,2^*^*^*, Sally Hargreaves ^2,3^, and Pippa Oakeshott ^2^

*^1^* Primary Care Unit, Department of Public Health and Primary Care, University of Cambridge, Cambridge, UK

*^2^* St George’s School of Health and Medical Sciences, Population Health Research Institute, City St George’s, University of London, London, UK

*^3^* The Migrant Health Research Unit, Institute for Infection and Immunity, City St George’s, University of London, London, UK

*^*^*Corresponding Author:

Primary Care Unit, Department of Public Health and Primary Care, University of Cambridge, East Forvie Building, East Forvie Site, Robinson Way, Cambridge, CB2 0SR, UK

[msr37@cam.ac.uk](mailto:msr37@cam.ac.uk)

**Challenges and Opportunities of Vaccination during Pregnancy: Perspectives of 20 Healthcare Professionals**

**Running title:** Vaccination in pregnancy

Mohammad S Razai ^ab^*^*^*, Sally Hargreaves *^bc^*, and Pippa Oakeshott *^b^*

*^a^* Primary Care Unit, Department of Public Health and Primary Care, University of Cambridge, Cambridge, UK

*^b^* St George’s School of Health and Medical Sciences, Population Health Research Institute, City St George’s, University of London, London, UK

*^c^* The Migrant Health Research Unit, Institute for Infection and Immunity, City St George’s, University of London, London, UK

*^*^*Corresponding Author:

Department of Public Health and Primary Care, University of Cambridge, East Forvie Building, East Forvie Site, Robinson Way, Cambridge, CB2 0SR

[msr37@cam.ac.uk](mailto:msr37@cam.ac.uk)

**Data and Methods**

***Online survey***

In Table 1 we provide the online survey among healthcare professionals involved in vaccination during pregnancy.

**Table S1: Vaccination in pregnancy: Healthcare professional online survey**

| **1.Age group (years)**  20-30  30-40  40-50  50-60  Over 60  Prefer not to say |
| --- |
| **2.Gender**  Woman  Man  Non-binary  Prefer not to say |
| **3.Ethnicity**  White British  Black African  Black Caribbean  Asian or Asian British  Other  Prefer not to say |
| **4.Professional Category**  Midwife  Obstetrician  Pharmacist  Nurse  General Practitioner  Other |
| **5.Place of Work**  Hospital  General Practice  Community Midwife  Community Pharmacy  Other |
| 6. Based on your experience, what challenges and barriers have you faced in both administering and promoting vaccinations during pregnancy? |
| 7. In your opinion, what are the most effective strategies to enhance vaccination rates during pregnancy? Please elaborate. |
| 8. Do you have any additional insights or recommendations regarding vaccination during pregnancy? |
| End of Survey  Submit |

**Table S2: Discussion guide prompts for healthcare professionals workshop**

| *Opening Prompt:* |
| --- |
| - Can you share your initial thoughts on the current state of vaccination uptake among pregnant women within your practice? |
| *Evolving Nature of Vaccine Uptake:* |
| - How have you seen attitudes towards vaccination in pregnancy change over time, and what do you believe are the driving forces behind these changes? |
| *Differences in Vaccine Acceptance:* |
| - In your experience, what are the most common concerns that pregnant women express about vaccines? |
| - How do vaccine acceptance rates vary among different communities or demographic groups that you serve? |
| *Impact of Communication and Messaging:* |
| - What messaging strategies have you found to be most effective when discussing vaccination with pregnant women? |
| - How do you address misinformation or concerns about vaccine safety and efficacy? |
| *Accessibility and Convenience of Vaccine Services:* |
| - Can you describe the challenges that pregnant women might face in accessing vaccination services? |
| - What improvements can be made to make vaccine services more convenient for pregnant women? |
| *Role of Healthcare Professionals:* |
| - What role do you think healthcare professionals should play in a pregnant woman’s decision to get vaccinated? |
| - How can midwives, in particular, be better supported to facilitate vaccine discussions and decisions? |
| *Influence of Family and Social Networks:* |
| - In what ways do you observe family and social networks influencing pregnant women's vaccination decisions? |
| - How can positive influences from these networks be harnessed to improve vaccine uptake? |
| *Use of AI and Digital Tools:* |
| - What potential do you see for AI and digital tools to impact vaccine decision-making among pregnant women? |
| - Have you had experience with any digital tools that support vaccination decisions? If so, what has been the feedback? |
| *Closing Prompt:* |
| - Reflecting on our discussion, what key strategies would you prioritise to improve vaccination rates among pregnant women? |
| *Additional Comments:* |
| - Is there anything else you would like to add that we have not covered, particularly regarding your personal experiences or insights into vaccination during pregnancy? |

*Ethical procedures*

The workshop and anonymous survey were exempt from ethics review in line with Medical Research Council (MRC) Criteria [Figure S1 below]. All participants were informed about the purpose of the study, and verbal informed consent was obtained before the workshop. Although the workshop was exempt from formal ethical approval, we adhered to ethical guidelines regarding data collection, consent, and the handling of recordings. Survey respondents provided written informed consent. Participants were assured of anonymity and the confidentiality of their contributions.


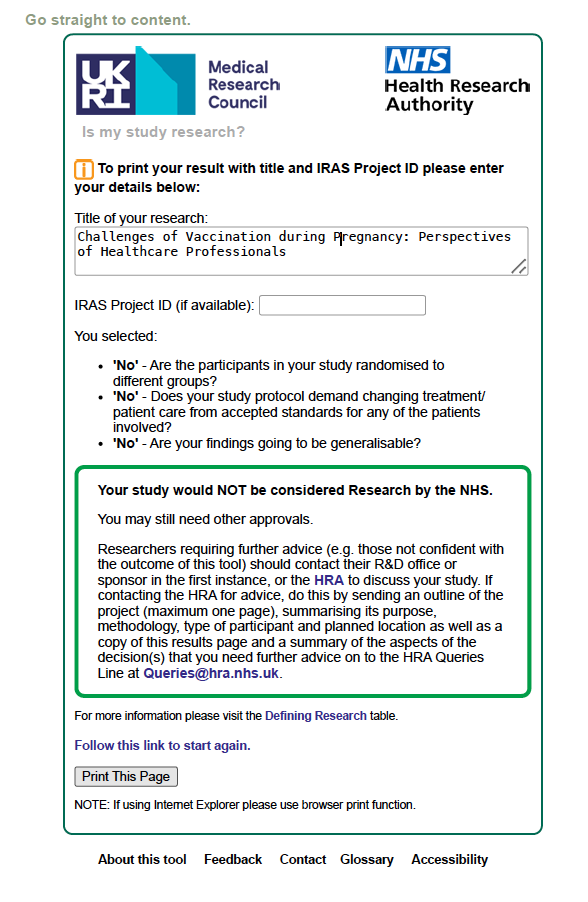


**Figure S1: MRC Criteria for Research**
